# Genome Analysis of the Multidrug-Resistant *Campylobacter coli* BCT3 of the Sequence Type (ST) 872 Isolated from a Pediatric Diarrhea Case

**DOI:** 10.3390/microorganisms13061420

**Published:** 2025-06-18

**Authors:** Konstantinos Papadimitriou, Anastasios Ioannidis, Aleksandra Slavko, Genovefa Chronopoulou, Nektarios Marmaras, Anastasia Pangalis, Elisavet Olntasi, Niki Vassilaki, Efthymia Ioanna Koufogeorgou, Iris Kolida, Dimitrios Theodoridis, Stylianos Chatzipanagiotou

**Affiliations:** 1 Laboratory of Food Quality Control and Hygiene, Department of Food Science and Human Nutrition, Agricultural University of Athens, Iera Odos 75, 11855 Athens, Greece; elisauetol@gmail.com; 2 Laboratory of Clinical Microbiology, School of Medicine, Attikon University Hospital, National and Kapodistrian University of Athens, Rimini 1, 12462 Chaidari, Greece; tasobi@med.uoa.gr; 3 Department of Food Science and Technology, School of Agriculture and Food, University of the Peloponnese, 24100 Kalamata, Greece; a.slavko@go.uop.gr; 4 Central Laboratory, Athens Medical Center, Distomou 5-7, 15125 Marousi, Greece; g.chronopoulou@iatriko.gr (G.C.); n.marmaras@iatriko.gr (N.M.); a.pangalis@iatriko.gr (A.P.); 5 Molecular Virology Laboratory, Department of Microbiology, Hellenic Pasteur Institute, Vasilissis Sofias 127, 11521 Athens, Greece; nikiv@pasteur.gr (N.V.); euthimianna@yahoo.gr (E.I.K.); iriskolida@gmail.com (I.K.); 6 Department of Clinical Microbiology, Athens Medical School, Aeginition Hospital, 11528 Athens, Greece; dimdrteo@gmail.com (D.T.); schatzipa@gmail.com (S.C.)

**Keywords:** *Campylobacter coli*, multidrug resistance, genome, virulence, antimicrobial resistance genes, pediatric, diarrhea, fluoroquinolones, macrolides

## Abstract

*Campylobacter jejuni* and *Campylobacter coli* are the two main campylobacter species that cause foodborne campylobacteriosis. Recent studies have reported that *Campylobacter* spp. are prone to developing resistance to antibiotics commonly used for their treatment, with many *C. coli* strains identified as multidrug-resistant. This study presents the results of the whole-genome sequencing analysis of the multidrug-resistant *C. coli* strain BCT3 isolated in Greece from a stool specimen of a pediatric patient presenting with diarrhea. The strain was isolated using selective culture media and, based on antimicrobial susceptibility tests, was found to be resistant to ciprofloxacin, tetracycline, erythromycin, azithromycin, clarithromycin, and doxycycline. To further characterize it, we performed whole-genome sequencing, which identified strain BCT3 as *C. coli*. Moreover, multilocus sequence typing assigned the BCT3 to the sequence type (ST) 872, belonging to clonal complex ST-828. The presence of multiple virulence genes revealed its pathogenic potential. The detection of antimicrobial resistance genes and mutated alleles was indicative of its resistance to fluoroquinolones, macrolides, and tetracyclines, supporting the observed phenotype. To our knowledge, this is the first reported clinical case of such a multidrug-resistant *C. coli* strain in Greece.

## 1. Introduction

*Campylobacter* is a major cause of bacterial gastroenteritis worldwide [1] and is associated with multiple gastrointestinal and neurological disorders, including inflammatory bowel disease (IBD), colorectal cancer, and Guillain–Barré syndrome [2,3,4]. Although *Campylobacter* infection can have a fatal outcome in children, the elderly, and immunocompromised individuals [5], the majority of cases do not require any treatment, as the disease is self-limited and the symptoms are mild [6]. *Campylobacter jejuni* and *Campylobacter coli* are the two most commonly detected species in humans, as compared to *Campylobacter lari* and *Campylobacter upsaliensis* [5].

The main route of *Campylobacter* transmission is through the consumption of raw or undercooked meat, especially poultry, while other transmissive sources can include the soil, water, and contact with domestic or wild animals [7,8]. It is widely acknowledged that chicken meat is a significant source of *Campylobacter* infections in humans on a global scale. *Campylobacter* spp. have been observed to colonize the intestinal tract of avian hosts in substantial numbers. Also, the transmission of these bacteria among flocks can result in elevated infection rates within the poultry population. At the level of the slaughterhouse, improper handling of chickens can result in carcasses becoming contaminated with *Campylobacter* from the intestinal content. The occurrence of cross-contamination is a potential hazard, with the transmission of bacteria from contaminated poultry products to water sources or food being a possibility [9]. It has also been demonstrated that due to the failure to comply with strict safety requirements during the slaughtering process, pig and cattle carcasses can become contaminated with *Campylobacter* [10]. Additionally, the bacterium was found at the highest rates in the neck region of the subjects in comparison with other anatomical regions. This could possibly be due to elevated exposure of these regions to the contents of the intestinal tract during the processing stage [11]. A study conducted in Greece in 2020 revealed that the prevalence of *Campylobacter* spp. in carcasses was found to be 70.42% positive for the presence of the bacterium. Furthermore, 73.94% of the cecum-based samples were found to be positive for *Campylobacter* spp. [12].

Distinctive genetic virulence factors of human *Campylobacter* isolates are the *cadF* and *flaA* genes. The *cadF* gene encodes the CadF adhesive fibronectin and facilitates adhesion to host cells. FlaA, the flagellin protein that is encoded by the *flaA* gene, is a part of the bacterial filament and is important for *Campylobacter* mobility [13,14,15]. The *flaA* locus is a short variable region (SVR) that is used in *Campylobacter* genotyping and phylogenetic analyses [16,17]. Among other significant virulence indicators that are associated with better host invasion are the *ciaB* and *virB11* genes [13].

The invasion of the intestinal tract by *Campylobacter*, particularly the crypts, is established by specific adhesion to the host’s epithelium proteins, accompanied by the colonization of intestinal cells. This is followed by *Campylobacter* proliferation and secretion of toxins, necrotizing the intestinal villi, resulting in damage to the epithelium of the intestines through the opening of the tight junctions and the shielding barrier, the release of electrolytes, and the initiation of inflammatory responses, resulting in severe and bloody diarrhea [18]. *Campylobacter* has been reported to induce the secretion of cytokines and Interleukin-8. Another bacterial protein used for adhesion and host invasion is JlpA, a lipoprotein of the surface that also binds to Hsp90α. This interaction activates both NF-κΒ and mitogen-activated protein (MAP) kinase, leading to proinflammatory responses [19].

The first-line treatment against *C. jejuni* and *C. coli* includes the administration of antimicrobials from the macrolide class (erythromycin, clarithromycin, and azithromycin) and tetracycline and its derivative doxycycline, and antimicrobials of the quinolone class (ciprofloxacin) [17,20]. Other treatment options for persistent campylobacteriosis cases are aminoglycosides (gentamicin, kanamycin, etc.) and carbapenems (meropenem and imipenem) [21,22].

Over the past few years, the extensive use of drugs in livestock farming has contributed to the development of high resistance towards different groups of antimicrobials used against *C. jejuni* and *C. coli* [23]. According to the 2022 surveillance report of the European Centre for Disease Prevention and Control (ECDC), the highest rate of resistance to macrolides for *C. coli* (38.5%) was detected in Greece, followed by Portugal (26.9%) and Spain (19.3%) [24]. Additionally, the “European Union summary report on antimicrobial resistance in zoonotic and indicator bacteria from humans, animals and food in 2021–2022” revealed that in contrast with *C. jejuni*, *C. coli* had elevated multidrug-resistance (MDR) in 9.0% of human, 8.3% of broiler, 16.9% of fattening turkey, 39.3% of young calf, and 9.5% of fattening pig isolates [25].

The outcomes of the antimicrobial resistance (AMR) assessment of foodborne pathogens like *Campylobacter* spp. are deeply concerning, underscoring an immediate imperative for the establishment of an integrated surveillance system to restrain the overuse of antibiotics. A “One Health” approach is imperative to curtail the excessive utilization of antibiotics in the context of animal farming and to forestall the spread of AMR through the food supply chain. The implementation of the “One Health” concept in the context of poultry farming necessitates a multifaceted approach and effective collaboration between food production, human health, and regulatory bodies. This could include enhanced biosecurity measures, more rational antibiotic usage, and more robust food safety regulations [11]. Relevant to these concepts, in the current study, we present the genome sequence of a multidrug-resistant *C. coli* strain, isolated from the stool of a 3-year-old male patient hospitalized with diarrheal symptoms.

## 2. Materials and Methods

### 2.1. Strain Isolation and Antimicrobial Susceptibility Testing

A strain of *Campylobacter coli* was isolated from a child’s diarrheic stool sample using *Campylobacter* Agar (Oxoid, Basingstoke, UK) that contains the antibiotics vancomycin, polymyxin B, amphotericin B, and trimethoprim to inhibit the growth of competing intestinal flora and allow for the selective isolation of *Campylobacter* spp. For culture, a *Campylobacter* agar plate was inoculated and incubated under microaerophilic conditions (5% O_2_, 10% CO_2_, and 85% N_2_) at 42 °C for 40–48 h. After the incubation period, the plates were examined for the presence of *Campylobacter* spp. Colonies with characteristic morphology were presumptively identified and further confirmed using the VITEK^®^ MS PRIME system (bioMérieux, Craponne, France), a matrix-assisted laser desorption/ionization time-of-flight mass spectrometry (MALDI-TOF MS) system, following the manufacturer’s instructions and established protocols [26]. Antimicrobial susceptibility testing was performed according to the European Committee on Antimicrobial Susceptibility Testing (EUCAST) standardized disk diffusion method [27]. Mueller–Hinton agar supplemented with 5% defibrinated horse blood and 20 mg/L β-NAD (Bioprepare, Keratea, Greece) was used as the testing medium. The bacterial inoculum was adjusted to a 0.5 McFarland turbidity standard using a densitometer (DensiCHEK Plus, bioMérieux, Craponne, France), according to EUCAST recommendations. The plates were incubated under microaerophilic conditions at 42 °C for 24 h. Inhibition zones were measured and interpreted in accordance with EUCAST guidelines [28].

### 2.2. Genome Sequencing and Bioinformatics Analysis

Genomic DNA of *C. coli* strain BCT3 was extracted using the MagCore Nucleic Acid Extraction Kit for bacteria and the respective equipment DNAex-MagCore-02 (RBC Bioscience Corp., New Taipei City, Taiwan). Whole-genome sequencing (WGS) of the BCT3 strain was performed by Novogene (Novogene, Cambridge, UK) on an Illumina NovaSeq 6000 platform (Illumina, San Diego, CA, USA) following standard procedures, including library preparation and 2 × 250 bp paired-end sequencing. After sequencing, the raw reads of the BCT3 strain were assessed for their quality with FastQC v0.11.9 [29] and trimmed using Cutadapt v4.5 [30]. Subsequently, the trimmed reads were assembled de novo using the Unicycler v0.5.1 assembly pipeline [31], and the quality of the obtained assembly was checked with Quast v5.0.2 [32]. Species identification of the BCT3 strain was conducted with the Type (Strain) Genome Server (TYGS) [33]. In order to obtain the reference-guided assembly, the initial assembly was further processed using the assembly tool Ragout v2.3 [34] with the *Campylobacter coli* reference genome for strain FDAARGOS_735 (GenBank accession number: CP046317.1). The completeness of the final reference-guided genome assembly was checked with Busco v5.0.0 [35], and its alignment against the chromosomal sequence of the reference strain was obtained using the Circoletto suite [36]. The genome map of *C. coli* BCT3, visualizing the genomic features and labeling of genes and proteins, was acquired with DNAPlotter [37]. A search to identify plasmid sequences was conducted using PlasmidFinder [38], and variant calling was carried out with Snippy v4.3.6 [39]. Multilocus sequence typing (MLST) and core genome MLST analysis were conducted with CLC genomics workbench v24.0.2 (Qiagen, Hilden, Germany) with the *C. jejuni*/*C. coli* MLST [40,41] and core genome *C. jejuni*/*C. coli* MLST (cgMLST) v1.0 schemes [42] using the PubMLST database [43]. The functional annotation of the final genome was made using the Rapid Annotation Subsystem Technology (RAST) server [44]. The presence of virulence genes in the *C. coli* BCT3 genome was explored using CLC genomics workbench v24.0.2 as well as the ABRicate v1.0.1 pipeline [45] through CamPype v1.0 [46] against the virulence factor database (VFDB) [47]. The antimicrobial resistance (AMR) of the *C. coli* BCT3 strain was explored using CLC genomics workbench v24.0.2 and CamPype v1.0, ABRicate v1.0.1 and AMRFinderPlus v3.11.2 [48] against ResFinder [49], the Comprehensive Antibiotic Resistance Database (CARD) [50], the ARG-ANNOT peptide marker database [51], MEGARes 2.0 [52], and the NCBI [48]. Only genes with alignment coverage exceeding 90% and sequence identity above 80% were considered as present in the genome [53,54].

## 3. Results and Discussion

### 3.1. General Characteristics of the Genome of Campylobacter coli Strain BCT3

**Figure 1:**
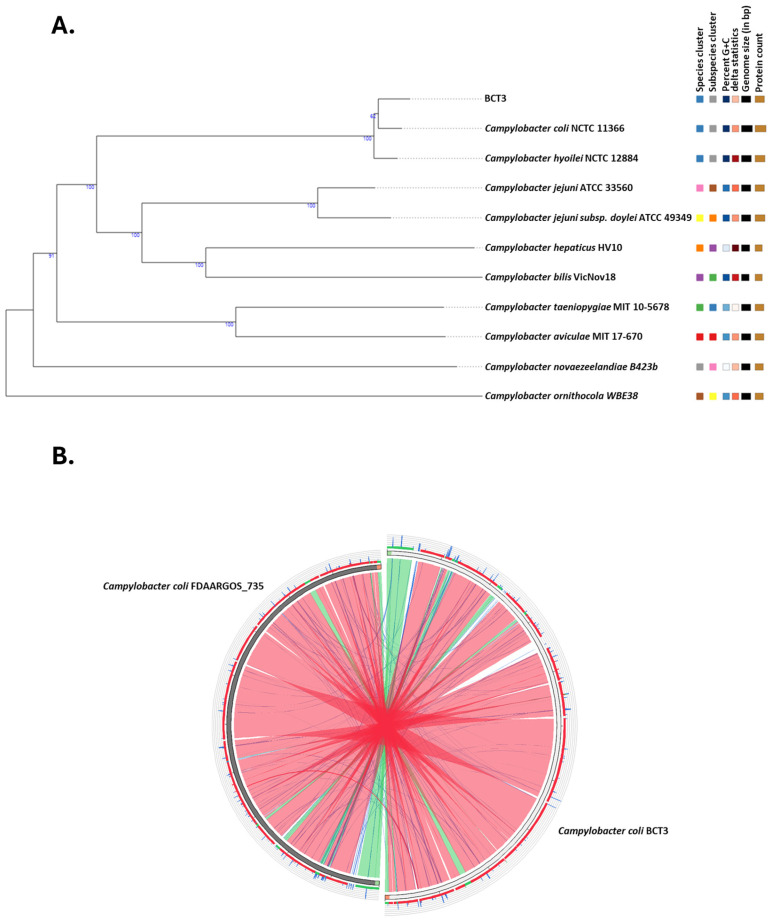
Phylogenetic and comparative genomic analyses of *Campylobacter coli* BCT3. Phylogenetic tree showing the relationship among the *C. coli* BCT3 and type strains in the TYGS database (**A**). Circular genome alignment between the *C. coli* BCT3 strain and the *C. coli* reference strain FDAARGOS_735 (**B**). Colored ribbons correspond to different levels of % identity according to BLASTn local alignment (red > 98%, green ≤ 98%, blue ≤ 95%, and orange ≤ 90%).

A high-quality de novo assembly of the genome under investigation was obtained, comprising 44 contigs with a total length of 1,690,752 bp and a GC% content of 31%. A total of 11 contigs exceeded 50,000 bp, and the assembly showed strong continuity with an N50 value of 162,311 bp and an L50 of 4. Genome analysis using the TYGS server identified strain BCT3 as *Campylobacter coli*, which agreed with the results of the Vitek MS Prime system (Figure 1A).

A reference-guided assembly produced a single scaffold of 1,731,905 bp in length with a number of unplaced contigs of a total length of 14,884 bp (0.87% of the total) and a completeness of 99.8%. Furthermore, alignment of *C. coli* BCT3 against the reference genome of strain FDAARGOS_735 revealed significant similarity, with most regions being syntenic (Figure 1B). Variant calling revealed a total of 6094 variants in the *C. coli* BCT3 genome compared to the reference sequence, out of which 4744 were accounted as Single Nucleotide Polymorphisms (SNPs). In addition, a small number of insertions (70) and deletions (52) were observed, suggesting minimal structural disruption in terms of added or missing nucleotides. Finally, no plasmids were identified according to the in silico analysis.

**Figure 2:**
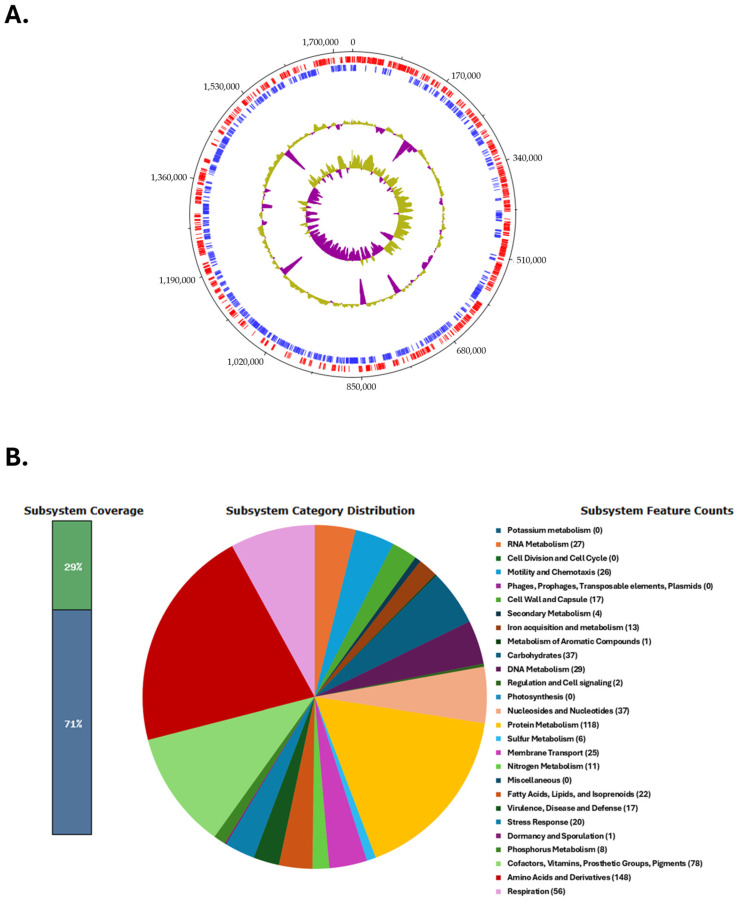
Genome characteristics of the *Campylobacter coli* BCT3 strain. Circular genome map of the *C. coli* BCT3 strain generated using DNAPlotter (**A**). Tracks from inside to outside represent the GC skew, GC content, and reverse (blue) and forward (red) coding sequences (CDSs). Functional categorization of the protein-encoding genes of the *C. coli* BCT3 genome assigned to subsystem categories (**B**).

The circular genome map of the *C. coli* BCT3 strain is shown in Figure 2A. The analysis of the protein-encoding genes of strain BCT3 with RAST showed that 29% of the annotated features were categorized into subsystems, while 71% of proteins could not be assigned to subsystem categories. The results indicated that the genome of the isolated strain harbors genes associated with “Amino acid and protein metabolism” (148 counts), “Protein metabolism” (118 counts), and “Cofactors, vitamins, prosthetic groups, pigments” (78 counts), among others (Figure 2B).

### 3.2. Multilocus Sequence Typing

**Figure 3:**
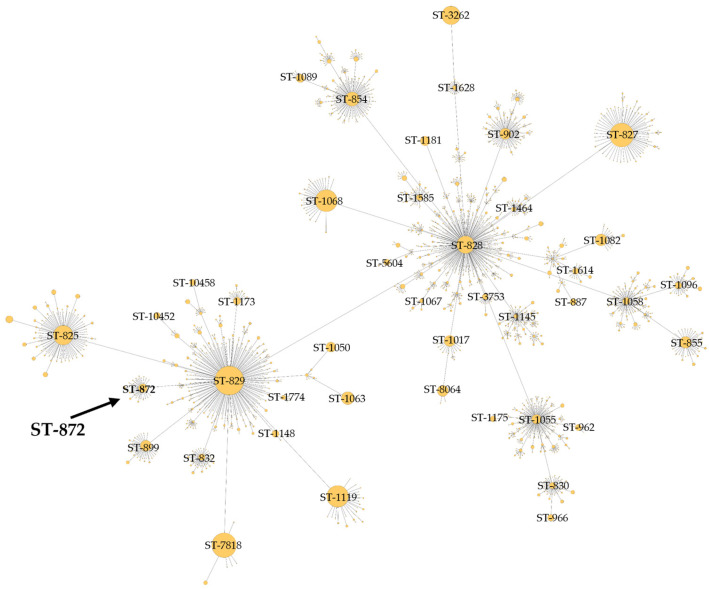
Minimum spanning tree of clonal complex ST-828 constructed for *C. coli* BCT3 according to the *C. jejuni*/*C. coli* MLST scheme.

The BCT3 genome was analyzed with the *C. jejuni*/*C. coli* MLST scheme targeting seven housekeeping genes (*aspA*, *glnA*, *gltA*, *glyA*, *pgm*, *tkt*, and *uncA*) [40,41,55]. The results obtained indicated that the BCT3 strain possessed *aspA* (33), *glnA* (39), *gltA* (30), *pgm* (113), *glyA* (82), *tkt* (44), and *uncA* (17) and was assigned to sequence type (ST) 872 belonging to the ST-828 clonal complex (CC) (Figure 3). Isolates of *C. coli* assigned to ST-872 have been identified in different hosts, predominantly in chickens [56,57,58,59,60,61,62,63] and humans [58,64,65,66,67,68,69,70], but also in geese [61] and swine [71]. It has also been suggested that *C. coli* of ST-872 is a potential foodborne risk for human infection through chicken consumption [56].

The genome of *C. coli* BCT3 was also subjected to analysis using the *Campylobacter jejuni*/*C. coli* core-genome MLST (cgMLST) scheme [42] and it was assigned to cgST 19335, but inconclusively.

### 3.3. Virulence Factors

The pathogenic potential of the *C. coli* BCT3 strain was further investigated. Our analysis revealed the presence of multiple genes that contribute to the virulence of *Campylobacter* spp. by facilitating adhesion, invasion, motility, chemotaxis, and evasion of the host innate immune system [72,73,74,75]. Adhesion and invasion genes included *cadF* and *pebA* (Table 1). The *cadF* gene encodes an outer membrane protein that binds fibronectin, promoting, in this way, adhesion to host epithelial cells and enabling colonization [74,76]. The *pebA* gene encodes a bifunctional protein that serves both as an adhesin and the substrate-binding component of an ABC transporter for aspartate and glutamate, contributing to host cell adhesion [77]. The invasion-associated genes *ciaB* and *ciaC*, which mediate the interaction of the pathogen with host cells, were also detected [72]. Genes encoding core structural components of the flagellum included *flgB*, *flgC*, *flgD*, *flgE*, *flgF*, *flgG*, *flgH*, *flgI*, *fliD*, *fliE*, *fliF*, *fliG*, *fliL*, *flaC*, *flaD*, and *flaG*. Of note, a partial *flaA* was detected after manual investigation, most probably due to a local assembly incompleteness. Genes responsible for motor function, secretion, and export comprised *pflA*, *motA*, *flhA*, *flhB*, *flhF*, *flhG*, *fliI*, *fliP*, *fliQ*, *fliR*, and *fliS*, while additional genes contributing to flagellar assembly and structural components included *flgJ*, *flgK*, *flgM*, *flgP*, *flgQ*, *fliW*, and *fliY* [72,74,75,78,79,80,81]. Chemotaxis-related genes involved in host colonization, *cheA*, *cheV*, *cheW*, and *cheY*, were also found, further increasing the virulence potential of the *C. coli* BCT3 strain [72,82,83]. Furthermore, we identified the presence of the two-component regulatory system genes *flgS* and *flgR*, along with *rpoN*, which encodes σ^54^, indicating the potential for σ^54^-dependent regulation of flagellar gene expression [72,84]. Additional regulatory genes included *fliA*, which encodes the flagellar-specific sigma factor σ^28^, and the motor switch proteins *fliM* and *fliN*, involved in controlling flagellar rotation [72,79]. Genes involved in flagellin glycosylation and post-translational modification, including *pseA*-*pseI*, *ptmA*, *ptmB*, and *eptC*, were also detected, supporting the completeness of the pseudaminic acid biosynthesis pathway and indicating the potential of alternative glycosylation mechanisms essential for flagellar function [59,79,85,86,87].

Other virulence genes detected were those involved in lipooligosaccharide (LOS) biosynthesis that play a significant role in the pathogenicity of *Campylobacter* spp. [72]. Genes *gmhA* and *gmhB*, involved in the biosynthesis of heptose sugars, an important component of LOS, were found to be present. Genes *waaC* and *waaF* encoding heptosyltransferases that add heptose sugar molecules to the LOS core structure were also detected [72,88,89,90,91,92]. Furthermore, gene *waaV* contributing to LOS core assembly was found along with *hldD* and *hldE*, which are associated with LOS biosynthesis [59,93]. The presence of all of these genes indicates a fully functional LOS biosynthetic pathway. Finally, genes protecting the *C. coli* BCT3 strain from host immune responses related to capsule biosynthesis and export included *kpsD*, *kpsF*, *kpsM*, *kpsS*, and *kpsT* [72,92]. Several of the aforementioned virulence genes have also been reported for *C. coli* strains of ST-872 [57,59,61,63].

**Table 1:** Virulence genes found in the *C. coli* BCT3 strain.

Gene	Function	Ref.
*cadF*	Outer membrane fibronectin-binding protein	[57,74]
*pebA*	Binding protein of an ABC transporter system	[77,94]
*ciaB*	Invasion antigen	[72,74]
*ciaC*	Invasion antigen	[72,74]
*flgB*	Flagellar basal body rod protein	[79,94]
*flgC*	Flagellar basal body rod protein	[79]
*flgD*	Flagellar basal body rod modification protein	[79]
*flgE*	Flagellar hook protein	[72,79,95]
*flgF*	Flagellar basal body rod protein	[80]
*flgG*	Flagellar basal body rod protein	[74]
*flgH*	Flagellar L-ring protein precursor	[80]
*flgI*	Flagellar P-ring protein precursor	[96]
*flgJ*	Flagellar protein involved in peptidoglycan hydrolysis	[96]
*flgK*	Flagellar hook-associated protein	[79]
*flgM*	Negative regulator of flagellin synthesis	[97]
*flgP*	Flagellar motility protein	[72]
*flgQ*	Flagellar motility protein	[75]
*flgR*	σ^54^—associated transcriptional activator	[74]
*flgS*	Signal transduction histidine kinase	[74]
*flhA*	Flagellar biosynthesis protein	[72,74]
*flhB*	Flagellar biosynthesis protein	[74]
*flhF*	Flagellar biosynthesis regulator	[79]
*flhG*	ATP-binding protein	[79]
*flaC*	Secreted flagellin	[72]
*flaD*	Flagellar protein	[81]
*flaG*	Flagellar filament length control	[72,97]
*fliA*	Flagellar biosynthesis sigma factor	[72]
*fliD*	Flagellar hook-associated protein	[79,98]
*fliE*	Flagellar hook–basal body complex protein	[79]
*fliF*	Flagellar M-ring protein	[74,79]
*fliG*	Flagellar motor switch protein	[74,79]
*fliI*	Flagellum-specific ATPse	[79]
*fliL*	Flagellar basal body protein	[79]
*fliM*	Flagellar motor switch protein	[79]
*fliN*	Flagellar motor switch protein	[79]
*fliP*	Flagellar biosynthesis protein	[74]
*fliQ*	Flagellar biosynthesis protein	[74]
*fliR*	Flagellar biosynthetic protein	[74]
*fliS*	Flagellar secretion chaperone	[72,78,79]
*fliW*	Flagellar assembly protein	[72,78]
*fliY*	Flagellar motor switch protein	[74]
*rpoN*	RNA polymerase factor σ^54^	[84]
*pflA*	Paralysed flagellum protein	[74]
*motA*	Flagellar motor protein	[72,74]
*cheA*	Histidine kinase sensor	[72,74,82,83]
*cheV*	Phosphotransferase	[72,74]
*cheW*	Phosphotransferase	[72,74]
*cheY*	Cytoplasmic response regulator	[72,74]
*pseA*	Pseudaminic acid biosynthesis protein	[87]
*pseB*	UDP-N-acetylglucosamine 4,6-dehydratase	[87,99]
*pseC*	UDP-4-amino-4,6-dideoxy-N-acetyl-beta-L-altrosamine transaminase	[87]
*pseD*	Motility accessory factor	[87]
*pseE*	Motility accessory factor	[79,87]
*pseF*	Acylneuraminate cytidylyltransferase	[87]
*pseG*	UDP-2,4-diacetamido-2,4,6-trideoxy-beta-L-altropyranose hydrolase	[87]
*pseH*	UDP-4-amino-4, 6-dideoxy-N-acetyl-beta-L-altrosamine N-acetyltransferase	[87]
*pseI*	N-acetylneuraminic acid synthetase	[100]
*eptC*	Phosphoethanolamine transferase	[59]
*ptmA*	Flagellin modification protein	[79,86]
*ptmB*	Acylneuraminate cytidylyltransferase	[79,86]
*gmhA*	Phosphoheptose isomerase	[91,92]
*gmhB*	DD-heptose 17-bisphosphate phosphatase	[91]
*waaC*	Heptosyltransferase I	[90,92]
*waaF*	Heptosyltransferase II	[92]
*waaV*	Glucosyltransferase	[92,93]
*hldD*	ADP-glyceromanno-heptose 6-epimerase	[59]
*hldE*	Bifunctional D-beta-D-heptose 7-phosphate kinase/D-beta-D-heptose 1-phosphate adenylyltransferase	[59]
*kpsD*	Capsule polysaccharide export system periplasmic protein	[72,92]
*kpsF*	D-arabinose 5-phosphate isomerase	[92]
*kpsM*	Capsule polysaccharide export system inner membrane protein	[72,92]
*kpsS*	Capsule polysaccharide modification protein	[92]
*kpsT*	Capsule polysaccharide export ATP-binding protein	[92]

### 3.4. Antimicrobial Resistance

Laboratory tests of the *C. coli* BCT3 strain showed its high resistance to multiple antibiotics, including ciprofloxacin, tetracycline, erythromycin, azithromycin, clarithromycin, and doxycycline (Table 2).

The observed MDR exerted by strain BCT3 could be attributed to the presence of AMR genes and mutated alleles (Table 3), which were detected in the genome sequence. These genes included *tet(O)*, one of the most prevalent resistance genes in *Campylobacter* spp. [101], which confers resistance to tetracycline, doxycycline, and minocycline [102,103] and bla_OXA-61_, bla_OXA-489_, and bla_OXA-605_, contributing to resistance against beta-lactam [104,105].

The analysis also showed the existence of aminoglycoside resistance genes, including *ant(6)-Ia*, *aad9*, and *aadE* [110,114]. Additionally, mutations *gyrA* (T86I) and *23S* (A2075G) were found, providing resistance to quinolones and macrolides, respectively [79,107,110,113]. Furthermore, a major multidrug efflux pump, CmeABC, which expels toxic compounds and plays a key role in *Campylobacter* resistance to a wide range of structurally diverse antimicrobials, was identified [107,111,115,116,117]. The full functionality of the CmeABC efflux pump requires the presence of all three components [107]. In addition, the *cmeR* gene, which encodes the transcriptional repressor CmeR regulating cmeABC expression, was also detected, albeit with 76.7% identity to the reference sequence [107,111]. This reduced identity may suggest a potential alteration in the regulatory function of CmeR, which could lead to the overexpression of CmeABC and contribute to increased antimicrobial resistance in the *C. coli* BCT3 strain [118,119]. Finally, another multidrug efflux pump, CmeDEF, was also present [74,107]. Although it may differ in function and in capacity to expel antibiotics and other toxic compounds from cmeABC, the presence of both of these systems in the *C. coli* BCT3 strain may enhance its antimicrobial resistance and adaptability to stresses [74,107,111,112].

It should be emphasized that other strains belonging to ST-872 within the ST-828 clonal complex isolated from human and chicken hosts have been found to be multidrug resistant (resistant to more than three different antimicrobial classes) [25,59,61,66,67]. These findings highlight the need for continuous monitoring and further assessment of the antibiotic resistance, virulence, and epidemiological behavior of strains belonging to this sequence type.

## 4. Conclusions

This investigation of the genome of the *C. coli* BCT3 strain isolated in Greece from a pediatric diarrhea case has provided important insights into its genetic composition, virulence potential, and antimicrobial resistance. The analysis revealed that the *C. coli* BCT3 strain belonged to ST-872 of clonal complex ST-828. Isolates of this type have been previously associated with human and chicken infections [57,59,61,63,67]. The identification of virulence-associated genes contributing to *C. coli* BCT3 strain immune evasion and intestinal colonization of the host revealed the pathogenic potential of this strain. Laboratory tests and analysis of AMR genes of the BCT3 strain validated its resistance to multiple antibiotics, including fluoroquinolones, macrolides, and tetracyclines. The MDR of the BCT3 strain, particularly its resistance to macrolides and fluoroquinolones, which are commonly used for the treatment of human campylobacteriosis, highlights potential challenges for clinical treatment [120,121]. The genome analysis reveals several characteristics of the strain. Nevertheless, further functional studies are needed to uncover novel aspects of its pathogenicity and antimicrobial resistance, particularly under simulated food processing or clinical conditions. Our findings emphasize the need to study multidrug-resistant strains of *C. coli* to understand the underlying resistance mechanisms and develop effective treatments for *Campylobacter* infections. The properties of the isolated multidrug-resistant strain BCT3 highlight the necessity for implementing suitable measures, ranging from livestock farming to health systems. Overall, this necessity arises from the concept of “One Health”, which emphasizes the interconnection between the health of humans, animals, plants, and the environment.

## Figures and Tables

**Table 2:** Resistance of the *C. coli* BCT3 strain to different antibiotics.

Antibiotic	Assessment *
Ciprofloxacin	R
Tetracycline	R
Erythromycin	R
Azithromycin	R
Clarithromycin	R
Doxycycline	R

* R: resistant. Resistance was chosen among other characterizations, including susceptible (S), susceptible increased exposure (I), and insufficient evidence (IE).

**Table 3:** Antimicrobial resistance genes found in the *C. coli* BCT3 strain.

Gene	Function	Resistance	Ref.
*tet(O)*	Tetracycline resistance protein TetO	Tetracycline, doxycycline, minocycline	[61,94,103,106,107,108]
*bla*_OXA-61_	Beta-lactamase	Beta-lactam	[104,105,106,108]
*bla*_OXA-489_	Beta-lactamase		[108,109]
*bla*_OXA-605_	Beta-lactamase		[61,81,106]
*bla*_OXA-450_	Beta-lactamase		[106]
*aad9*	Aminoglycoside nucleotidyltransferase	Aminoglycoside	[61,106]
*ant(6)-Ia*	Aminoglycoside nucleotidyltransferase		[79,107,110]
*aadE*	Aminoglycoside 6-adenylyltransferase		[61,106,107]
*cmeA*	RND efflux system membrane fusion protein CmeA	Multidrug-resistance	[74,106,107,111,112]
*cmeB*	Multidrug efflux RND transporter permease subunit CmeB		
*cmeC*	Multidrug efflux transporter outer membrane subunit CmeC		
*cmeD*	Multidrug efflux RND transporter outer membrane subunit CmeD	Multidrug-resistance	[74,107,112]
*cmeE*	Multidrug efflux RND transporter periplasmic adaptor subunit CmeE		
*cmeF*	Multidrug efflux RND transporter permease subunit CmeF		
*gyrA T86I*	DNA gyrase subunit A (T86I)	Fluoroquinolone	[61,113]
*23S A2075G*	23S rRNA mutation (A2075G)	Macrolide	[61,113]

## Data Availability

The whole-genome shotgun project has been deposited in GenBank under the bioproject ID PRJNA1237805.
